# Human mesenchymal stromal cells ameliorate complement induced inflammatory cascade and improve renal functions in a rat model of ischemia-reperfusion induced acute kidney injury

**DOI:** 10.1371/journal.pone.0222354

**Published:** 2019-09-12

**Authors:** Shani Zilberman-Itskovich, Ramzia Abu-Hamad, Rina Zarura, Marina Sova, Yafit Hachmo, Moshe Stark, Sara Neuman, Shimon Slavin, Shai Efrati

**Affiliations:** 1 Nephrology Division, Assaf-Harofeh Medical Center, Zerifin, Israel; 2 Sackler School of Medicine, Tel-Aviv University, Tel-Aviv, Israel; 3 Biotherapy International, The Center for Innovative Cancer Immunotherapy & Regenerative Medicine, Weizmann Center, Tel Aviv, Israel; National Institutes of Health, UNITED STATES

## Abstract

**Introduction:**

The primary rational for using mesenchymal stromal cells (MSCs) to rejuvenate damaged tissue is mostly based on their capacity to trans-differentiate and repair injured organs. However, previous studies have demonstrated that MSCs are beneficial even at very early stages, before differentiation and proliferation can be expected. The aim of the current study was to investigate the multifaceted immunological effects of systemically administrating MSCs in the setting of acute kidney injury (AKI) induced by ischemic-reperfusion (I/R).

**Methods:**

A rat model of I/R induced AKI was used. The rats underwent a unilateral nephrectomy with simultaneously clamping the contralateral kidney for 60 minutes. Four treatment groups received intravenously, increasing doses of human MSCs and after 48 hours, the rats were sacrificed. Blood was taken to evaluate renal functions and to measure systemic inflammatory markers. Kidneys were taken for histopathologic examinations and evaluations of intra-renal complement activation and inflammatory mediators.

**Results:**

Renal functions improved in U shaped dose dependent manner. Mean serum creatinine levels were 4.5, 2.9, 2.6, 1.7 and 4.1 mg/dL in I/R + placebo, I/R + 150x10^3^ cells, I/R + 250x10^3^ cells, I/R + 500x10^3^ cells and I/R + 1,000x10^3^ cells respectfully (p-values<0.05). Urea demonstrated consistent results with the same U shape improvement manner. The extensive activation of the complement system was ameliorated in the MSCs treatment groups. In addition, MSCs significantly decreased intra-renal levels of IL-1β and TNF-α. It should be noted that the highest doses of MSCs induced renal hypoxia, marked by the Hypoxy-probe staining.

**Conclusions:**

The early beneficial effect of MSCs in the setting of AKI may be attributed to their immunomodulatory effects. Safe treatment with MSCs can block the deleterious activation of the complement cascade and alleviate the hazardous inflammatory mediator-related cascade.

## Introduction

Acute kidney injury (AKI) is a common cause for morbidity and mortality with devastating long term consequences including end stage renal disease (ESRD) and dialysis dependence [1]. While AKI complications are effectively treated with dialysis, there is no clinical accepted specific treatment for preventing or reversing AKI damage [2]. One main mechanism responsible for AKI is ischemia-reperfusion (I/R) injury along with the resulting immunological consequences that include activation of the complement system and tubular damage [3, 4].

The use of mesenchymal stromal cells (MSCs), multipotent cells with self-renewal properties that can differentiate into mesodermal line cells, is one of the more promising AKI therapeutic approaches [5]. The primary rational for MSCs is that they can replace the damaged cells. However, there is growing evidence that early beneficial outcomes of MSCs therapy is attributed to their multifaceted immunological effects [5–7]. In experimental studies, following renal I/R injury, MSCs migrate to the injured site where they alleviate damage by secreting bioactive paracrine factors which generate a supporting environment that alleviates kidney damage [5]. However, the potential effects of MSCs on complement activation in I/R induced AKI has yet to be investigated.

The aim of the current study was to investigate the potential role of systemic administration of MSCs in I/R induced AKI. We wanted to gain a better understanding of their multifaceted immunological roles, including complement activation.

## Materials and methods

This study was strictly carried out according to the recommendations of the Guide for the Care and Use of Laboratory Animals of the National Institutes of Health. The protocol was approved by the Committee of Animal Experiment Ethics at Assaf-Harofeh Medical Center (Protocol Number: 25/2016), Israel. All surgeries were performed under halothane anesthesia, and all efforts were made to minimize suffering.

Seventy-three male Sprague–Dawley rats, eight-weeks old weighing 250-300g, were used. The rats were housed in animal cages at a temperature of 25°C with free access to food and water, in our institution’s animal facility.

### Ischemia-reperfusion model and treatment protocol

Rats were assigned to one of the following groups: (1) unilateral nephrectomy followed by an intravenous (IV) injection of saline 0.9% (control + placebo); (2) unilateral nephrectomy followed by IV injection of 1000x10^3^ MSCs (control + 1000x10^3^); (3) I/R followed by IV injection of saline 0.9% (I/R + placebo); (4) I/R followed by IV injection of 150x10^3^ MSCs (I/R + 150x10^3^); (5) I/R followed by IV injection of 250x10^3^ MSCs (I/R + 250x10^3^). (6) I/R followed by IV injection of 500x10^3^ MSCs (I/R + 500x10^3^); (7) I/R followed by IV injection of 1000x10^3^ MSCs (I/R + 1000x10^3^).

#### Surgical procedure

The rats were anesthetized with halothane under aseptic conditions. Unilateral nephrectomies were performed using an anterior approach laparotomy after clamping the right renal hilum. I/R was performed using an anterior approach laparotomy, and included unilateral nephrectomy of the right kidney, together with 60 minutes ischemia by clamping the left renal artery and vein followed by clamp removal and reperfusion. At the end of the procedure, the rats received an intravenous injection through the tail vein. Once recovered, the rats had free access to food and water. Urine was collected for evaluation.

### Mesenchymal stromal cells

Cells used in the present work were from Placentix Ltd. (Weizmann 14, Tel-Aviv, Israel, RN 514861202), a company that cryopreserves placenta & cord tissue derived cells. They have a consent signed by the families of each newborn baby, approving the use of some MSCs for basic research.

Protocol: Mesenchymal stem cells (MSCs) were isolated from human placentas which were donated after delivery. A written informed consent was obtained from the patient. Placenta tissue was excised and used for MSCs collection according to research protocols that were prepared in compliance with the International Society for Stem Cell Research (ISSCR) guidelines.

The placenta was repeatedly washed in phosphate-buffered saline (PBS, Biological Industries, Israel) and cut under a laminal flow hood into small pieces (<0.5 mm^3^) that were incubated in 30ml DMEM (Renium), 1% penicillin and streptomycin (Biological Industries) and 0.5% collagenase B (Sigma) and filtered to form a single cell suspension. The cells were maintained in an incubator inside 75 cm^2^ culture flasks (ThermoFisher scientific) for 48 hours in αMEM (Renium) containing 10% fetal bovine serum (Biological Industries), L-glutamine and 1% penicillin and streptomycin at 37ᵒC, 5% CO_2_. Media were changed every three days until the cell monolayer was around 70–80% confluent.

Flow cytometry characterization of MSCs indicated that cultured cells were positive for CD73 and CD29 with a lower expression of CD105, and negative for the leukocyte common antigens CD45, CD34 and CD31 (data not shown).

### Biochemical and immunological evaluations

The rats were sacrificed under anesthesia with halothane, 48 hours following the procedure. Blood was drawn via a cardiac puncture, and the left kidney was removed for pathological evaluation. The blood was centrifuged at 3,000 RPM for 10 minutes and serum was separated and tested for creatinine, urea; C-reactive protein (CRP), tumor necrosis factor (TNF)-α and interleukin (IL)-10 as systemic inflammation markers; C3, C4, classical and alternative pathways as markers of systemic complement activity.

The classical complement pathway, alternative complement pathway, C3 and C4 were assessed by specific ELISAs: HBT classical complement pathway, rat assay cat: HIT 410 (Hycult biotech, ENCO); HBT alternative complement pathway (Hycult biotech), rat cat: HIT 412; Rat C3 ELISA cat: E-25C3 (by ICL); C4 ELISA kit: rat complement 4; C4 ELISA kit MBS70336 (by MYBIO) according to the manufacturer’s instructions.

TNF-α (kit: RTA00), IL-10 (kit R1000) (all purchased from R&D Systems, USA), were also assessed from serum samples by specific ELISAs according to the manufacturer’s instructions.

### Pathological evaluation

Kidneys were preserved in 4% formalin and subsequently embedded in paraffin. Paraffin-embedded slides were prepared by a standard procedure. One slide from each rat was stained with hematoxylin-eosin dye for histopathologic examination under a light microscope. Another slide from each rat was used for immunofluorescence staining procedures according to the manufacturer’s instructions, for evaluating the mouse anti-rat complement C3 (NOVUS Biotest), and rabbit anti-C6 complement component 6 (Proteintech Biotest).

Computerized morphometry was performed using an Olympus CKX 41 microscope with the CMS-2-M system as part of the Advanced Measurement Systems, Ltd. (Israel). The system includes a digital color CCD camera (1600x1200 pixels) and a software package for pathology & immunohistochemistry evaluation.

Tubular necrosis was identified and calculated as the percentage of damaged tubules from the total of all tubules in the examined kidney [8]. In addition, nucleus degeneration and proliferation, and protein casts were evaluated and presented as % of tubules involved per total tubule count.

Complement C3 and C6 were subjectively evaluated by scoring between 0–5. A score of +5 was the highest immunofluorescence staining and 0 was no staining within the kidney.

To quantify the complement, we used the same immunofluorescence described above and quantifying the staining using ‘Lionheart FX Automated Live Cell Imager’ software (BioTek VT, USA) on the same slides.

#### Immunofluorescence staining for evaluating human cells

Mouse anti-human nuclei monoclonal antibody (MAB1281 Millipore Merck) was used for immunofluorescence staining procedures, according to the manufacturer’s instructions.

Ten randomly selected fields per each slide were examined, and the areas of interest were interactively selected by two independent observers, using proper optical threshold and microscope filter combinations. Computer morphometry was performed using Lionheart FX Automated Live Cell Imager (BioTek VT, USA). The system included a digital color camera and software package for immunofluorescence evaluation.

#### Immunofluorescence staining for evaluating inflammatory cytokines

IL-1β (MAB501-SP), IL-6 (AF506-SP), IL-10 (AF519-SP) and TNF-α (NBP2-34372-0.02mg) antibodies were used on the tissue (R&D Systems, USA), according to the manufacturer’s instructions. Microscopic quantification evaluation was done using Lionheart FX Automated Live Cell Imager (BioTek VT, USA).

#### Immunofluorescence staining for macrophages and apoptotic cells

Macrophage antibodies for CD 68 (KP1: sc-20060) and CD 163 (GHI/61: sc-20066) were used (Santa cruz biotechnology, USA) according to manufacturer’s instructions. Apoptosis was evaluated using BAX BCL2L4 (pro-apoptotic Bcl-2 protein, Proteintech 60267-1-g), and using ApopTag S7165 (Chemicon international) according to manufacturer’s instructions.

#### Intra-renal hypoxia

Intra-renal hypoxia was marked in vivo using a Hypoxyprobe TM-1 Plus kit (Chemicon, USA). In brief, 60mg/kg body weight of Hypoxyprobe-TM-1 in a 0.5ml bolus was injected via the caudal vein 1h prior to sacrifice. After sacrificing, the kidneys were collected and embedded in paraffin. FITC-conjugated Hypoxyprobe-1 Mab (Biotest) was used as the primary antibody. Microscopic evaluation for quantification was done using the Lionheart FX Automated Live Cell Imager (BioTek VT, USA).

### Statistical analysis

Data are presented as mean ± standard deviation for continuous data. Categorical descriptive data are presented as absolute values with percentages. Differences between groups were evaluated by Mann–Whitney and T-tests. A p-value of ≤0.05 was assumed to be significant. Data were analyzed by SPPS Version 25.0.

## Results

### Safety of MSCs treatment

**Fig 1:**
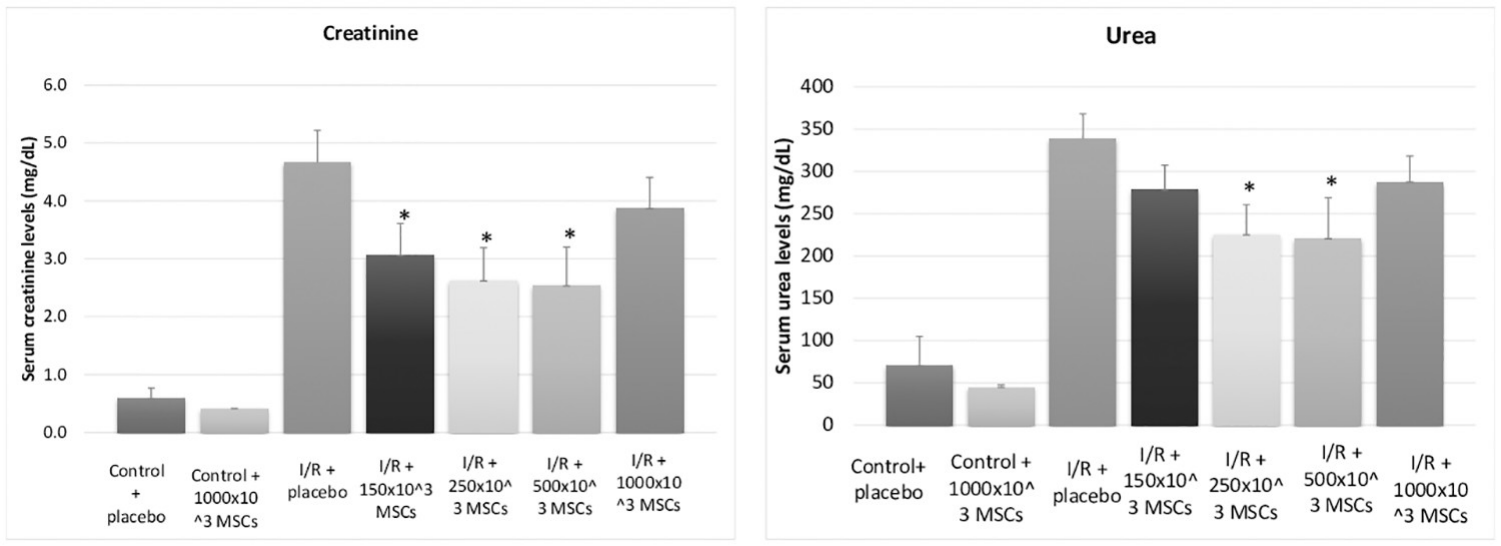
Renal function tests. Serum measurements of creatinine and urea levels of all groups, including control and ischemia-reperfusion groups, demonstrating a U-shaped response to mesenchymal stromal cells Abbreviations: I/R- ischemia reperfusion, MSCs- mesenchymal stem cells *p-value<0.05 for I/R + placebo compared with I/R + MSCs.

The safety of administrating 1000x10^3^ MSCs versus placebo was demonstrated in the two unilateral nephrectomies groups. No differences between the groups were observed regarding renal function tests, inflammation markers or pathological evaluation (Table 1 and Fig 1).

**Table 1:** Blood and urine tests for the control and I/R groups.

	Control + placebo	Control + 1000x10^3^ MSCs	I/R + placebo	I/R + 150x10^3^ MSCs	I/R + 250x10^3^ MSCs	I/R + 500x10^3^ MSCs	I/R + 1000x10^3^ MSCs
Blood tests							
**Hemoglobin (mean ± SD), g/dL**	12.5 ± 1.1	13.1 ± 0.8	12.4 ± 1.4	12.7 ± 1.3	12.3 ± 0.8	12.6 ± 0.8	12.2 ± 1.3
**White blood cells (mean ± SD), K/uL**	5.2 ± 3	3.9 ± 2.1	5.9 ± 2	4.8 ± 3.4	6.2 ± 3.5	5.6 ± 2.8	5.2 ± 2.4
**Platelets count (mean ± SD), K/uL**	850 ± 70	787 ± 75	746 ± 248	880 ± 231	789 ± 102	772 ± 88	688 ± 243
**Systemic renal function evaluation**							
**Creatinine (mean ± SD), mg/dL**	0.58 ± 0.41*	0.41 ± 0.02	4.67 ± 2.13*ᶧ	3.06 ± 2.21ᶧ	2.61 ± 1.83ᶧ	2.53 ± 2.24ᶧ	3.87 ± 2.15
**Urea (mean ± SD), mg/dL**	69.8 ± 78.5*	44.4 ± 6.6	338.5 ± 114.3*ᶧ	278 ± 167.7	224.8 ± 113.9ᶧ	220.2 ± 161.5ᶧ	287.2 ± 124.4
**Systemic inflammatory markers**							
**CRP (mean ± SD), mg/L**	0.31 ± 0.03	0.35 ± 0.08	0.33 ± 0.08	0.3 ± 0.0	0.38 ± 0.08	0.3 ± 0.0	0.35 ± 0.09
**IL-10 (mean ± SD), pg/mL**	43.3 ± 2.2	42.8 ± 1.1	43.9 ± 2.4	49.3 ± 16.9	42.1 ± 2.5	44.4 ± 1.76	42.9 ± 2.8
**TNF-α (mean ± SD), pg/mL**	18.3 ± 0.8*	19 ± 0.5	19.7 ± 1.1*	19.3 ± 1.0	19.9 ± 2.7	19.5 ± 1.3	19.6 ± 1.1
**C3 (mean ± SD), μcg/L**	54.8 ± 7.6	54.6 ± 52.2	49.6 ± 54.6	27.4 ± 22.4	49.6 ± 54.6	54.4 ± 55.2	20.5 ± 9.8
**C4 (mean ± SD), ng/L**	255 ± 74	325 ± 30	402 ± 72	460 ± 16	356 ± 67	395 ± 137	415 ± 84
**Classical complement (mean ± SD), % of activity**	71 ± 5	66 ± 5	69 ± 6ᶧ	61 ± 5ᶧ	70 ± 6	74 ± 8	68 ± 7
**Alternative complement (mean ± SD), % of activity**	84 ± 8	88 ± 13	72 ± 31	88 ± 13	92 ± 10	90 ± 7	81 ± 9
**Renal pathology**							
**Proliferation (% ± SD)**	58 ± 14	33 ± 14	18 ± 9*	25 ± 25	19 ± 6	38 ± 7	60 ± 13ᶧ
**ATN (% ± SD)**	33 ± 14	25 ± 5	75 ± 5*	63 ± 13	75 ± 10	31 ± 6ᶧ	70 ± 5
**Protein casts medulla (% ± SD)**	25 ± 5	15 ± 9	58 ± 14	63 ± 13	75 ± 18	19 ± 6ᶧ	80 ± 5ᶧ

MSCs = mesenchymal stromal cells, CRP = C-reactive protein, IL = interleukin, TNF = tumor necrosis factor, ATN = acute tubular necrosis

* p-value < 0.05 for Control + placebo versus I/R + placebo groups

^ᶧ^ p-value < 0.05 for I/R + placebo versus I/R + treatment group

### I/R induced AKI, inflammation and complement activation

#### Renal function tests

Compared to the control group, I/R induced acute renal failure, as measured by a significant increase in serum creatinine and urea (Table 1 and Fig 1).

#### Systemic inflammation results

Compared to the control group, TNF-α was significantly increased in the I/R group (p = 0.015). I/R did not affect the serum level of CRP, IL-10, C3 or C4s (Table 1).

#### Immunohistochemical results

I/R resulted in the intra-renal activation of the complement system as revealed by increased intra-renal deposits of C3 and C6 (Table 1).

### The effect of MSCs treatment on I/R induced AKI

#### Estimated renal function

**Fig 2:**
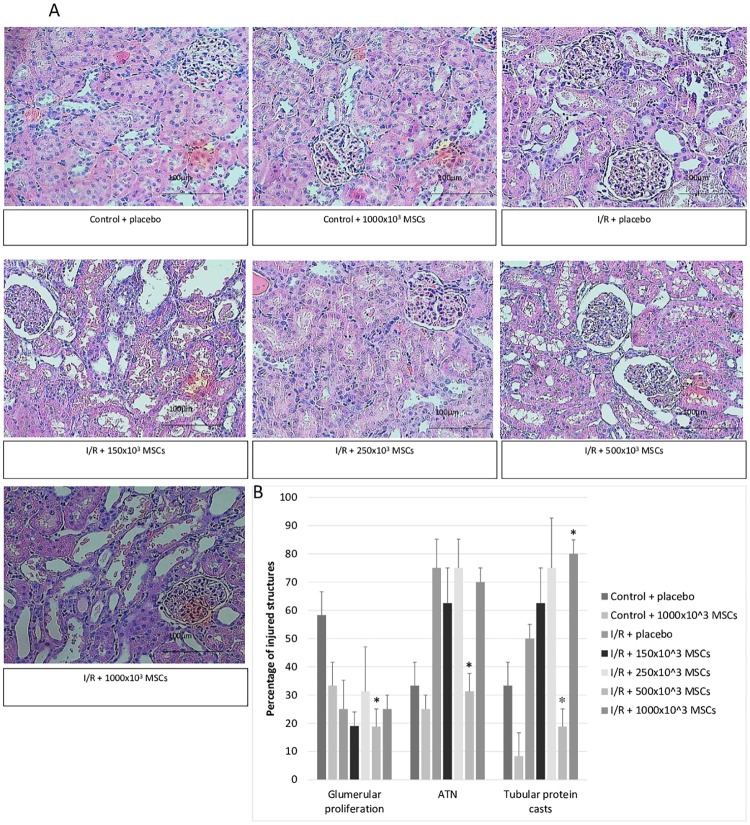
Histological evaluation. A- Hematoxylin & eosin staining pictures representing each control and treatment groups. B- Histological evaluation of control and ischemia-reperfusion groups demonstrating percentage of injured glomerular and tubules Abbreviations: ATN- acute tubular necrosis, I/R- ischemia reperfusion, MSCs- mesenchymal stem cells *p-value<0.05 for I/R + placebo compared with I/R + MSCs.

As detailed in Figs 1 and 2, and Table 1, eGFR measured by serum creatinine and urea improved in a u-shaped dose response curve in I/R + MSCs groups with the maximal beneficial effect seen in the 500x10^3^ injected MSCs group.

#### Renal pathology

As with eGFR, the maximal beneficial effect with regards to renal pathology was most noticeable the I/R group that received an injection of 500x10^3^ MSCs. The findings of the pathological evaluations are summarized in Table 1 and Fig 2.

#### Immunohistochemical results

**Fig 3:**
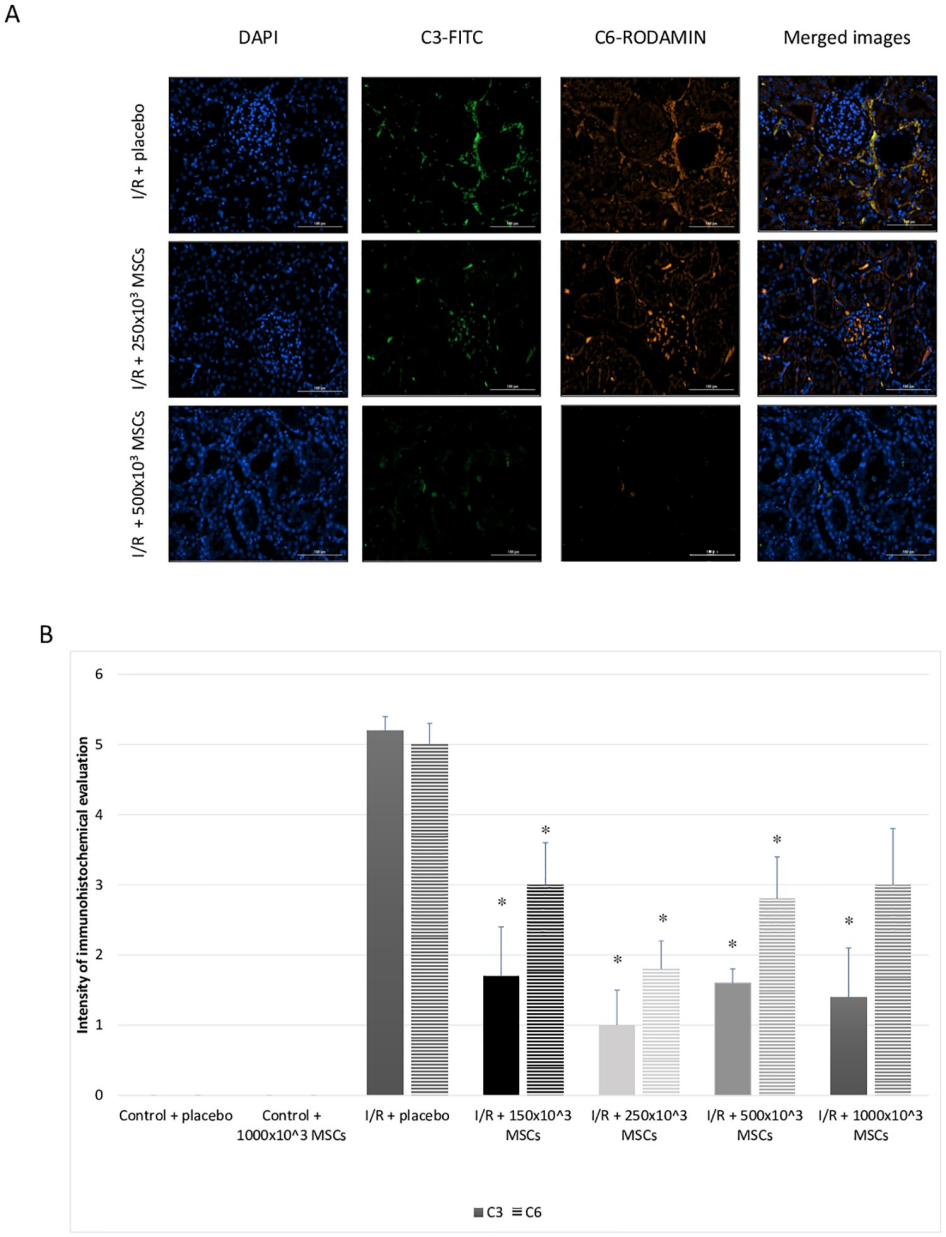
Immunohistochemical complement evaluation. A- figures of complement staining for different groups. B- quantification of complement C3 and C6 levels by intensity. Abbreviations: I/R- ischemia reperfusion, MSCs- mesenchymal stem cells *p-value<0.05 for I/R + placebo compared with I/R + MSCs.

**Fig 4:**
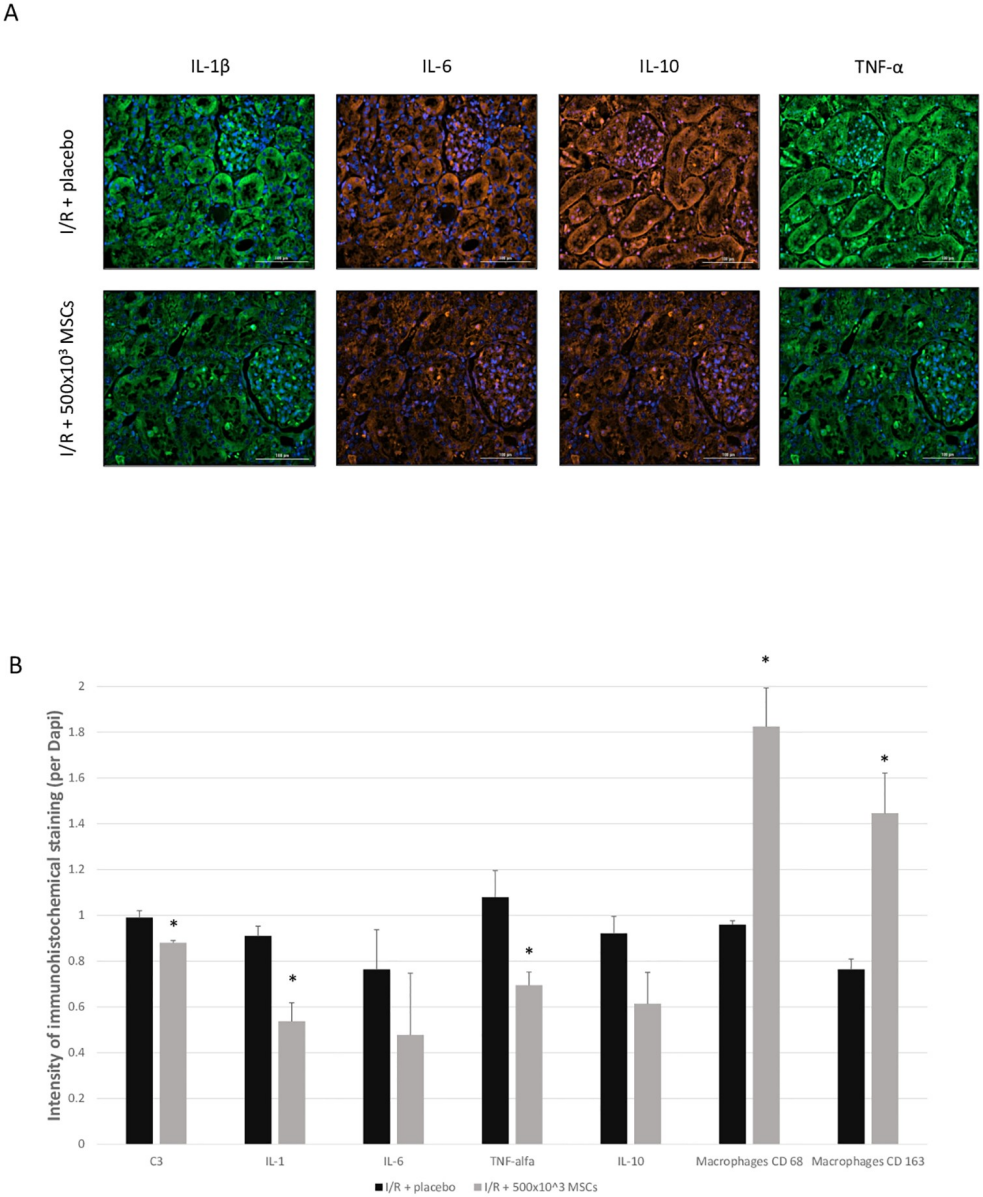
Tissue cytokines. A- intensity of intra-renal immunohistochemical staining demonstrating a reduction in cytokine levels. B- figures of cytokine staining for IL-1β, IL-6, IL-10, and TNF-α, comparing I/R + placebo and I/R + 500x10^3^ MSCs groups. The nucleolus is seen in blue, IL-1β and IL-6 staining are on the same slide, as are IL-10 and TNF-α. Abbreviations: I/R- ischemia reperfusion, MSCs- mesenchymal stem cells, TNF- tumor necrosis factor, IL- interleukin *p-value<0.05 for I/R + placebo compared with I/R + MSCs.

**Fig 5:**
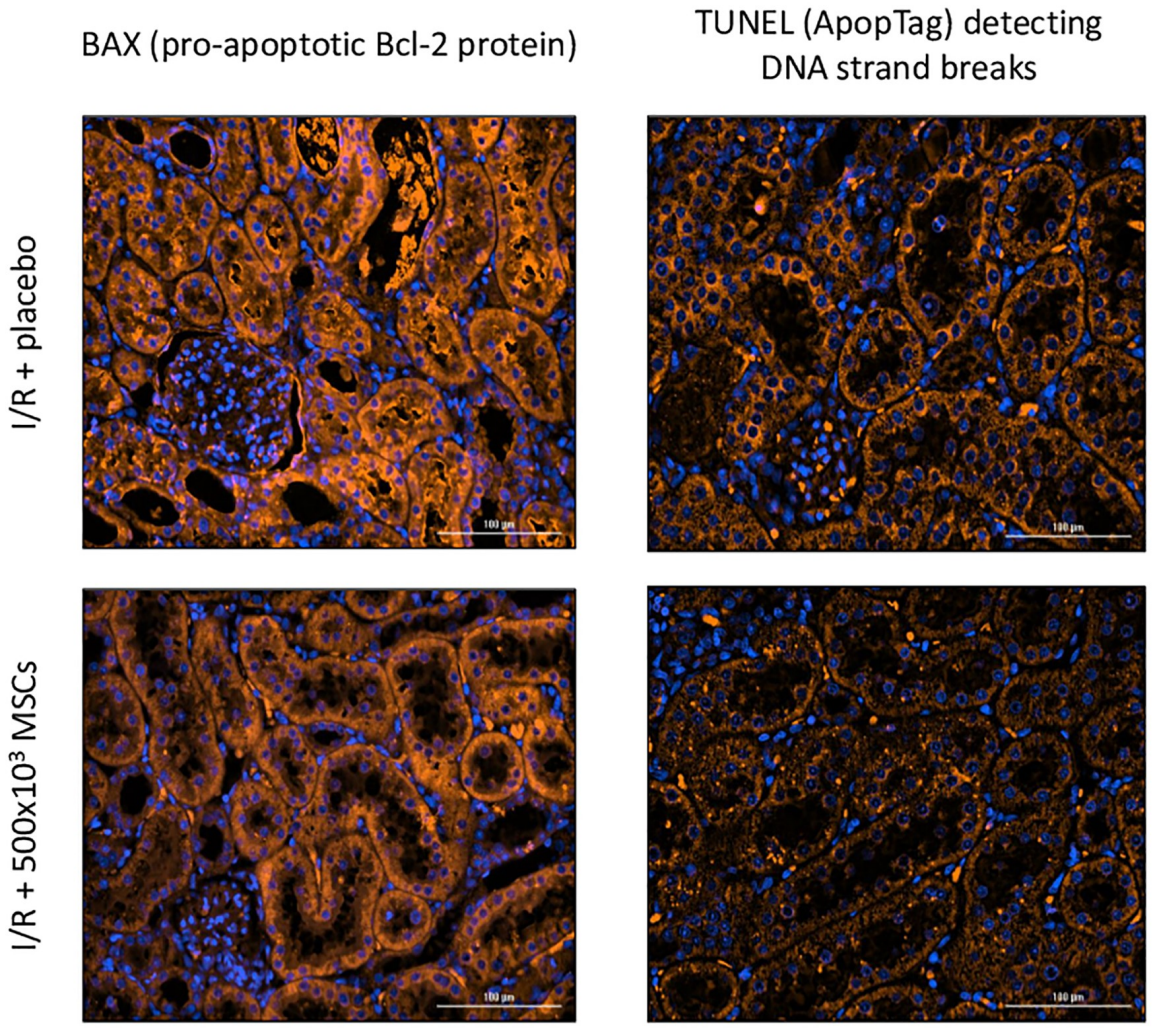
Apoptosis in I/R + placebo and I/R + MSCs treatment group. BAX pro-apoptotic Bcl-2 protein (orange) and TUNEL (ApopTag) detecting DNA strand breaks demonstrating less apoptosis in the treatment group, but without reaching statistical differences. The nucleolus is seen in blue. Abbreviations: I/R- ischemia reperfusion, MSCs- mesenchymal stem cells.

Compared to the I/R + placebo group, the expected increase in intra-renal complement C3 and C6 deposits was ameliorated in all the MSCs treated groups (Fig 3). Quantification of complement deposits revealed C3 intensity of 0.99 ± 0.07 for I/R + placebo group and 0.88 ± 0.03 for I/R + 500x10^3^ (p = 0.004). Additionally, MSCs significantly decreased intra-renal levels of IL-1β and TNF-α (p-value 0.01 for both groups) and they were similar to the control group. IL-6 and IL-10 were also lower in the 500x10^3^ MSCs treated group, but did not reach a statistically significant difference (Fig 4). Quantification of macrophages in the tissue demonstrated an increased macrophage presence after administration of MSCs: CD 68 was 0.96 ± 0.04 and 1.82 ± 0.34 for I/R + placebo group and I/R + 500x10^3^ MSCs groups respectively (p = 0.008). CD 163 was 0.76 ± 0.09 and 1.45 ± 0.39 for I/R + placebo group and I/R + 500x10^3^ MSCs groups respectively (p = 0.008). Apoptosis was evaluated using two different assays, the BAX pro-apoptotic Bcl-2 protein and TUNEL (ApopTag) detecting DNA strand breaks. Both assays demonstrated reduced apoptosis in the I/R + MSCs treatment group (intensity of 7.73E-06 and 1.82E-10) compared with the I/R + placebo group (intensity of 9.45E-06 and 2.58E-10) in BAX and TUNEL respectively. However, both assays did not reach statistically significant differences (p = 0.3 and 0.056 for BAX and TUNES respectively) (Fig 5).

#### Intra-renal MSCs

**Fig 6:**
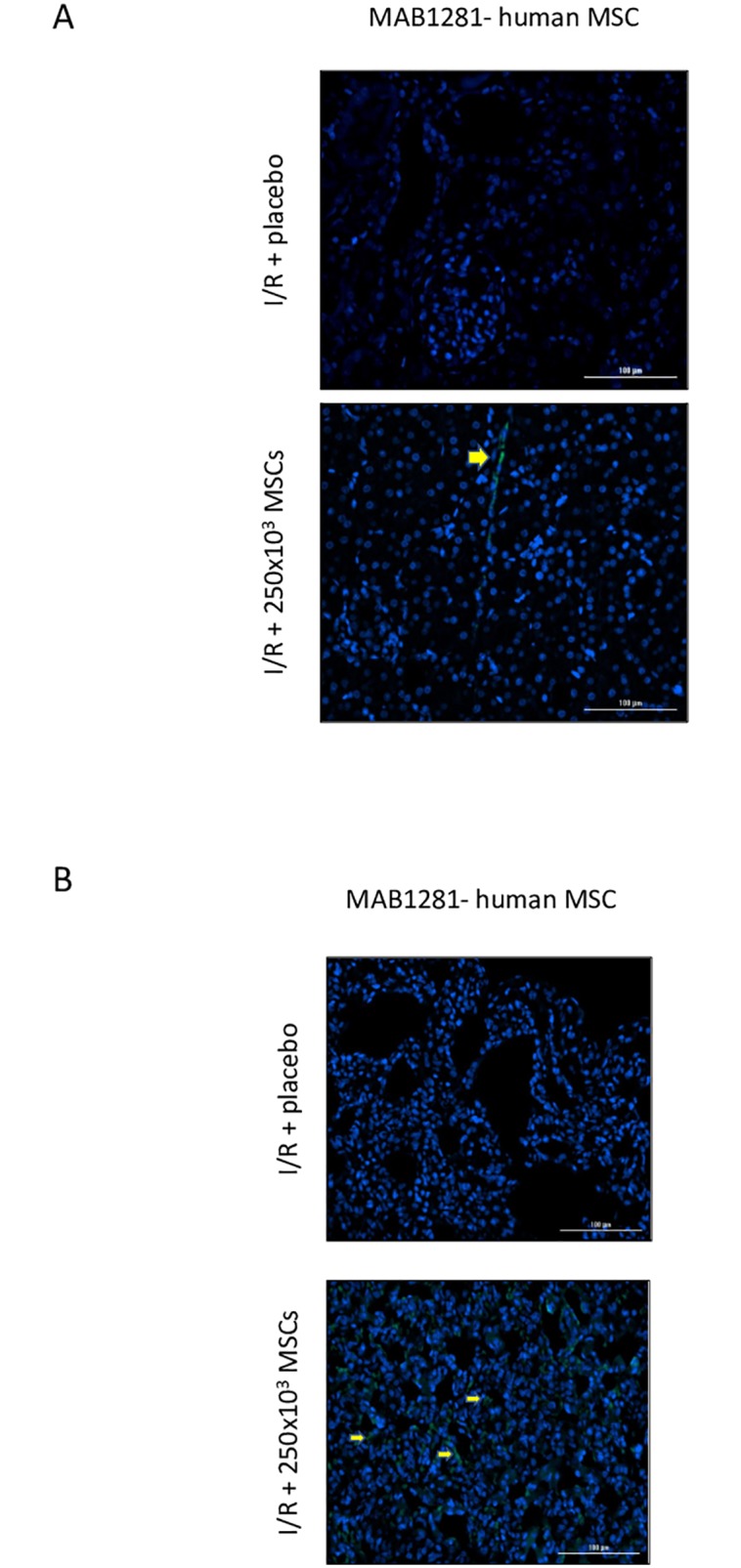
Human MSCs in rat tissue. A- a kidney demonstrating positive staining for MAB1281- humanized marker. B- a lung demonstrating positive staining for MAB1281- humanized marker. The nucleolus is demonstrated in blue. Abbreviations: I/R- ischemia reperfusion, MSCs- mesenchymal stem cells.

After systemic administration, the injected human MSCs were found mainly in the lungs, where they arranged in a linear network appearance. MSCs were also seen to a lesser degree in the I/R damaged kidneys where they had a linear network appearance (Fig 6). MSCs were not found in the liver, spleen or heart tissues.

#### Intra-renal hypoxia

**Fig 7:**
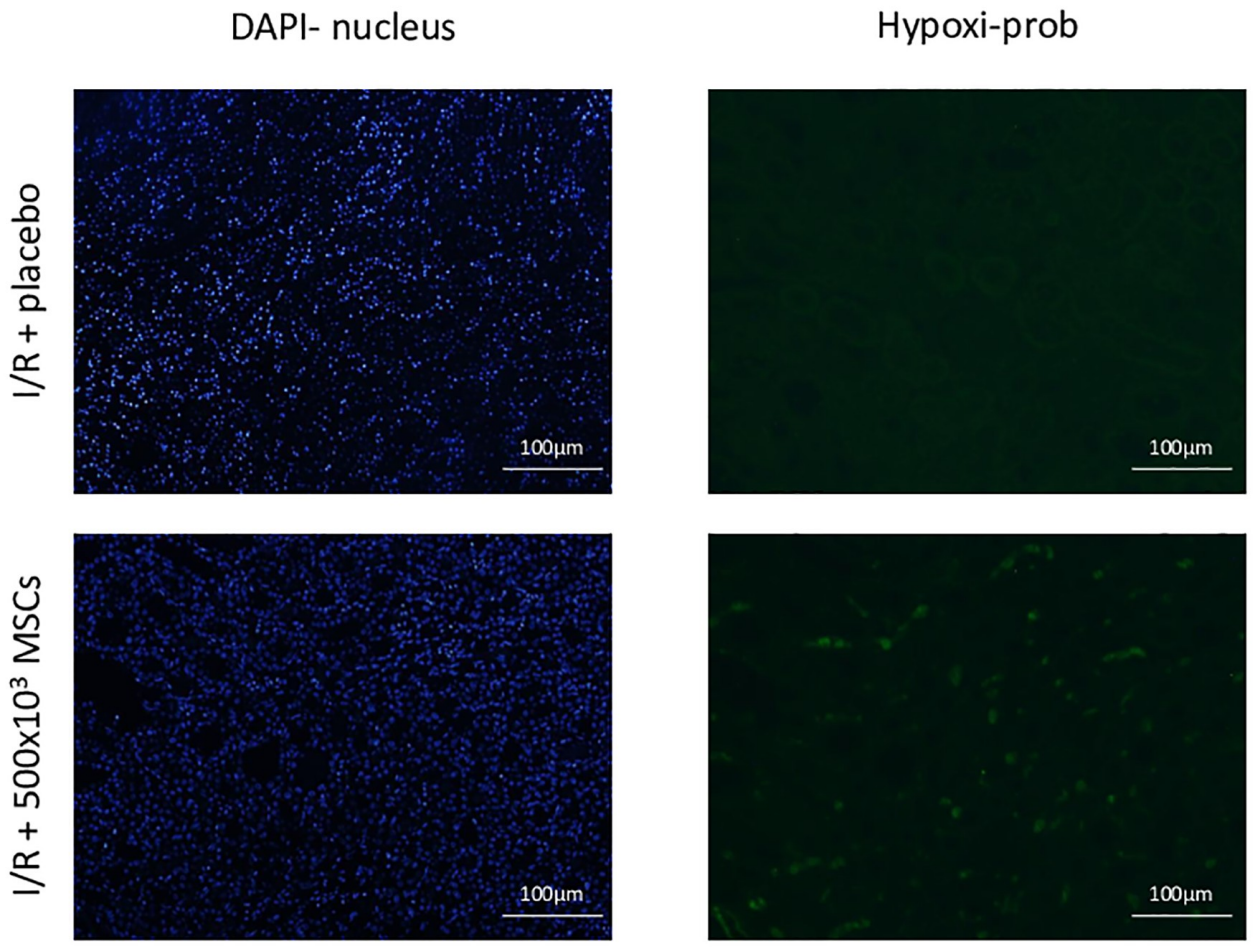
Tissue hypoxia. Staining intensity for the treatment groups Abbreviations: I/R- ischemia reperfusion, MSCs- mesenchymal stem cells.

Hypoxia was measured in all I/R kidneys as compared to the sham control. Hypoxia was higher in the I/R + 1000x10^3^ MSCs group compared to the I/R + placebo or to the lower MSCs doses. Quantification results demonstrated an intensity staining of 0.02 ± 0.02 for I/R + placebo and 0.05 ± 0.01 for I/R + 1000x10^3^ MSCs group (p = 0.09), (Fig 7).

## Discussion

In this current study, we investigated the systemic effects of intravenously administered MSCs on I/R induced AKI injury. Reperfusion injury after ischemia is characterized by a severe immune response, including elevated reactive oxygen species and an increase in pro-inflammatory cytokines, including IL-1β, IL-6 and TNF-α [3, 9, 10].

The results of this study demonstrate that the initial beneficial effects of MSCs on AKI can be attributed to blocking the intra-renal complement activation and intra-renal inflammatory responses. The improvement seen in the rats’ renal function 48h after AKI induction correlates well with MSCs-induced anti-inflammatory effects, since it is too early to be due to the potential intra-renal differentiation of MSCs. Additionally, high levels of IL-1β in the injured kidneys were almost eliminated after MSCs administration. This early effect was also seen in lungs by Ortiz et al. [11] who hypothesized that the protective effect of MSCs in lung injury is related to the suppression of IL-1 by IL-1-receptor antagonists released by the MSCs. A previous study also demonstrated immunomodulatory effects of MSCs both locally and systemically after AKI [12].

As expected [4], the intra-renal complement system was significantly activated after the reperfusion period. This activation was blunted after MSCs administration even at relatively low doses. Besides the pathologic effect of the activated complement system on the tubular cells, there is also a chemoattractant effect of C3a, C5a and C1q on MSCs [13, 14]. Tang et al. [15] showed the relationship between C5a and the C5a receptor and the protective effect of MSCs in a mouse AKI model.

Additionally, the complement cascade is also capable of inducing IL-1β production [16]. Asgari et al. [17] have shown that C3a is an important factor for producing IL-1β in monocytes, which in turn can induce inflammation in I/R injury and increase the tubular damage [9, 18]. In the current study, IL-1β as well as its related TNF-α intra-renal concentrations were both ameliorated by MSCs administration.

Macrophages have an important contribution to cytokine release in the tissue [19]. In our study, macrophages were substantially increased in the I/R group after administering MSCs cells. An increase of both CD68 and CD163 positive cells were detected in the tissue. Two types of macrophages are usually described in renal pathology: M1 macrophages that are considered pro-inflammatory cells and M2 macrophages that are considered anti-inflammatory cells [20]. While CD68 is used as a pan-macrophage marker, CD163 is a specific M2 marker [21]. In our trial, CD68/CD163 cells markedly increased after injecting MSCs, indicating an increase in anti-inflammatory M2 macrophages. This observation was also seen by Wise et al., who suggested that these macrophages promote regeneration after I/R [22]. In addition, it should be noted that macrophages have an important contribution to endogenous repair mechanisms after acute kidney injury, by several pathways, for example by secreting cytokines such as IL-22 and by providing ligands for retinoic acid (RA) and canonical Wnt-b catenin signaling [23].

Apoptosis is a form of cell death, that has previously demonstrated as part of the pathogenesis of I/R injury [24, 25]. In the current study, apoptosis was evaluated by two assays. Both assays revealed lower apoptosis rates in the MSCs treated groups, but statistical significance was borderline (0.056). Possibly, if apoptosis was evaluated earlier (the evaluation point in the current study protocol was 48 hours after the acute insult), the decline in apoptosis rate would have been more prominent.

There is an ongoing debate as to whether MSCs should be injected systematically or directly into the damaged organ. Different ways of administration were evaluated in different studies to alleviate I/R induce AKI. In a rat model, three main routes of administration are acceptable: intravenous (IV), intraarterial (IA) and intraperitoneal (IP) [12]. In this study we chose systemic IV administration to resemble the more practical way of potential future administration of MSCs in clinical practice. Most of the MSCs were found in the lungs and a smaller amount were found in the injured kidney, 48 hours after systemic IV administration. No MSCs were found in other organs including the liver, spleen and heart. Our results are in line with previous studies showing that MSCs migrate to the acutely injured kidney [22, 26]. MSCs can also home in to other injured tissues such as the heart and liver [27]. These observations support the homing capabilities of MSCs to the I/R injured kidney.

MSCs are currently being studied as therapeutic agents in many autoimmune diseases and other clinical conditions caused by inflammatory processes, as well as after kidney transplantation. As confirmed by previous investigations in preclinical [27, 28] and clinical studies [29], MSCs infusion is usually safe and well tolerated [5]. However, the U shape dose dependent response curve seen in the current study, including reduced beneficial effects at higher MSC doses, should be taken into consideration in any future clinical study. Our results indicate that the decrease in beneficial effects at higher dosages is attributed to worsening of renal hypoxia. A dose-dependent study was conducted for renal artery injection of MSCs after AKI [28]. Their study also showed that a dose of 1×10^5^ MSCs was significantly better than 1×10^6^ MSCs [28]. Further studies are needed to find the optimal cell-dose levels for optimal clinical effects in the clinical setting.

Although the animal model results are promising, there is no assurance of treatment success in patients with different forms of renal failure. A recent study done on patients after cardiac surgery, designed to shorten time of recovery after AKI, did not show promising results [29]. One possible explanation could be the timing of the MSCs administration. In most preclinical studies demonstrating the efficacy of MSCs, the administration of MSCs was performed immediately after the insult, before clinical evidence of AKI was apparent. However, in the cardiac surgery study, MSCs were administrated only after AKI was clinically evident. MSCs administration timing might be an important factor not only for treating established renal damage but also for the intra-renal complement system activation. Li et al. [30] demonstrated that MSCs are injured by activated proteins of the complement system.

**In conclusion**, the beneficial effect of MSCs on I/R induced AKI may be attributed to their multifaceted anti-inflammatory and immunomodulatory effects which probably stem from their cell-mediated effects, or paracrine effects mediated by secretory exosomes or extracellular vesicles. In any case, MSCs can block the deleterious activation of the complement cascade and alleviate the inflammatory mediator cascade. There is a U shape dose response curve and further studies are needed to address the optimal dosage use in clinical settings. We are currently comparing the therapeutic effects of MSCs against MSC-secretory nano-particles, exosomes and extracellular vesicles, to further study the anti-inflammatory effects of MSCs.
